# Isotopic evidence for anthropogenic lead exposure on a 17th/18th century Barbadian plantation

**DOI:** 10.1002/ajpa.23938

**Published:** 2019-10-16

**Authors:** Jason E. Laffoon, Kristrina A. Shuler, Andrew R. Millard, James N. Connelly, Hannes Schroeder

**Affiliations:** ^1^ Faculty of Archaeology Leiden University Leiden The Netherlands; ^2^ Department of Sociology, Anthropology, and Social Work Auburn University Auburn Alabama; ^3^ Department of Archaeology Durham University Durham UK; ^4^ Center for Star and Planet Formation The GLOBE Institute, University of Copenhagen Copenhagen Denmark; ^5^ Section for Evolutionary Genomics, The GLOBE Institute University of Copenhagen Copenhagen Denmark

**Keywords:** bioarchaeology, health, isotopes, lead, slavery

## Abstract

**Objectives:**

To identify and characterize anthropogenic lead sources on a 17th/18th century Barbadian plantation and to test if lead isotope analyses can be used to identify the geographic origins of first‐generation African captives.

**Materials and Methods:**

We carried out lead (Pb) isotope analyses on dental enamel samples from 24 individuals from the Newton Plantation Cemetery in Barbados, which had previously been analyzed for strontium (Sr) and oxygen (O) isotope composition (Schroeder et al., American Journal of Physical Anthropology, 2009, 139:547–557) and Pb concentrations (Schroeder et al., American Journal of Physical Anthropology, 2013, 150:203–209.

**Results:**

We are able to identify British Pb sources, and more specifically Bristol/Mendips Pb, as the most likely source of anthropogenic Pb on the plantation, highlighting the impact of the British Atlantic economy on the lives of enslaved peoples in Barbados during the period of plantation slavery. Furthermore, we find that there is only one clear outlier among seven individuals who had previously been identified as African‐born based on their enamel Sr isotope composition (Schroeder et al., American Journal of Physical Anthropology, 2009, 139:547–557). All other individuals present a very homogenous Pb isotope composition, which overlaps with that of British Pb sources.

**Conclusion:**

Our results indicate that while Pb isotope analyses can help identify and further characterize the sources of anthropogenic Pb in plantation settings, they might not be suited for identifying the origins of African‐born individuals in diasporic contexts.

## 1 INTRODUCTION

### 1.1 Lead poisoning in the colonial Caribbean

**Figure 1:**
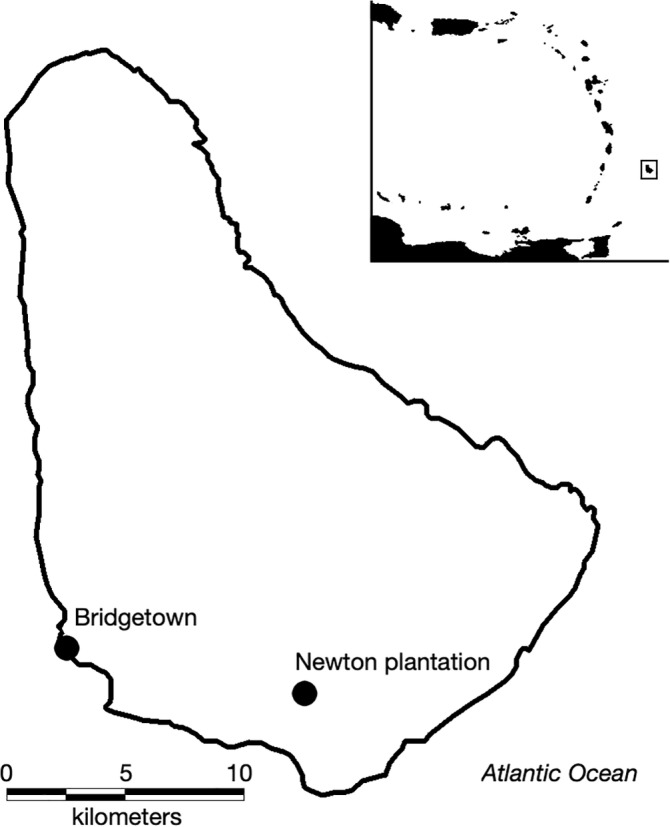
Map of Barbados showing the location of the Newton Plantation

Lead was used extensively on Caribbean plantations during the period of the transatlantic slave trade. The presence of lead had a devastating effect on the people living on the plantations, including children, and the effects of lead poisoning are well described in the historical record (Corruccini, Aufderheide, Handler, & Wittmers, 1987; Handler, Aufderheide, Corruccini, Brandon, & Wittmers Jr, 1986; Schroeder et al., 2013). Most information regarding lead exposure in enslaved populations comes from the Newton Plantation cemetery in Barbados (Figure 1), where 17th–18th century skeletal remains attest to frequent anthropogenic exposure. Lead toxicity can result from ingestion or inhalation of vapors, and colonial accounts provide widespread evidence of gastrointestinal illness (constipation, nausea, and loss of appetite), infertility, and neurosensory complications (lethargy, seizures, and weakness), which are clinically documented in cases of lead intoxication (Handler et al., 1986).

Various common household items were sources of lead contamination, including earthenwares, pewter drinking and serving vessels, flour, cosmetics, hair dyes, house paints, and medicaments. Water sources could become contaminated through lead‐lined cisterns and gutters, though less regularly available to the enslaved who collected water from nearby ponds (Handler et al., 1986). The most likely routes of exposure for the enslaved were, arguably, rum and molasses, which were contaminated through leaded fixtures and piping (Handler et al., 1986). Average bone lead content from Newton (*n* = 48; 117.6 ± 94.9 ppm or μg lead/g bone ash) was found to be nearly four times greater than reported for enslaved groups from the United States. (Aufderheide et al., 1985; Corruccini et al., 1987; Handler, 2006; Handler et al., 1986), and the lead levels of adults of both sexes appear to have resulted from chronic exposure throughout life (Corruccini et al., 1987). Schroeder et al. (2013) subsequently evaluated dental enamel in 26 individuals from the site, which, with earlier bone lead data, showed a clear pattern of chronic lead accumulation beginning at, or even before, birth.

Exposure during infancy and early childhood, as indicated by dental enamel, may have occurred in utero through endogenous maternal bone stores released into circulating blood, crossing the placenta (Ronchetti et al., 2006; Winecker, Ropero‐Miller, Broussard, & Hammett‐Stabler, 2002). However, the exceptionally high levels of lead observed in perinates are unlikely to have resulted solely from gestational release alone. Lead contamination may also have resulted from lactation (Gulson et al., 1998) and perhaps also from pica/geophagy, which is the craving and consumption of nonfood items: a practice that was documented archivally for the enslaved on the island (Handler, 2006) and a common route of contamination in children in industrialized nations today (Choi, Tanaka, Koren, & Ito, 2008).

### 1.2 Lead isotope analysis of skeletal remains for provenance research

Over the last several decades, there has been a substantial increase in the application of isotopic approaches for tracking modern and ancient human and animal movements (Makarewicz & Sealy, 2015). Isotope analyses of archeological skeletal remains for paleomobility and provenance studies have primarily focused on strontium (Sr) or oxygen (O) isotope systems. A recent trend has been the increasing use of multi‐isotope proxy data including both ^87^Sr/^86^Sr and δ^18^O, sometimes in conjunction with carbon (C) and nitrogen (N) isotope analyses. This trend reflects the widely acknowledged observation that there are inherent limitations of single isotope proxy data. One of the more confounding limitations is the issue of equifinality (Price, Burton, & Stoltman, 2007), whereby multiple locations possess similar isotope ranges making it impossible to distinguish between two or more possible places of origin. Owing to these persistent issues, increasing the number of analyzed isotopes may provide more reliable identifications of local versus nonlocal origins and potentially improve the accuracy of assessments of geographic place of origin (Laffoon et al., 2017). Lead (Pb) isotope analysis of human dental enamel arguably offers the greatest potential for contributing to paleomobility research owing to its high spatial variability and well documented geological and geographical constraints (Evans, Pashley, Madgwick, Neil, & Chenery, 2018).

The principles of the Pb isotope approach are comparable to the Sr isotope method (Bentley, 2006; Ericson, 1985; Price, Burton, & Bentley, 2002). Lead from the environment is ingested or inhaled and becomes incorporated into body tissues (Gulson, 2012; Kamenov & Gulson, 2014). It tends to be fairly quickly removed from soft tissues whereas the skeletal system holds the greatest proportion of the overall Pb load as Pb replaces Ca within bioapatite, the primary mineral component of teeth and bones (Gulson, 2012). Dental enamel, unlike bone and dentine, does not undergo subsequent remodeling, and therefore it preserves the isotope signal of the biochemical environment where it formed (Budd, Millard, Chenery, Lucy, & Roberts, 2004; Gulson & Gillings, 1997). As such, archaeological enamel Pb signatures can serve as geographic proxy indicators and provide information concerning an individual's childhood or natal origins (Budd et al., 2004; Carlson, 1996; Chiaradia, Gallay, & Todt, 2003; Montgomery, Evans, Powlesland, & Roberts, 2005).

There are four naturally occurring isotopes of Pb, of which ^204^Pb is not radiogenically produced whereas the other three isotopes ^206^Pb, ^207^Pb, and ^208^Pb are produced by the radioactive decay of other elements (^238^U, ^235^U, and ^232^Th, respectively) and, thus, are widely used for geochronology applications (Faure & Mensing, 2005). The relative abundance of the Pb isotopes to each other (Pb isotope ratios) in rocks and minerals is dependent on the original amounts of Pb, U, and Th in the parent material and its age. As such, geographic variation in natural environmental Pb isotope composition is primarily conditioned by geological variation (age and lithology of bedrock and sediments).

To date, applications of Pb isotope analyses of archeological human remains to address questions of paleomobility and provenance are comparatively rare, but such studies have become more common in recent years (Dudás, LeBlanc, Carter, & Bowring, 2016; Fitch, Grauer, & Augustine, 2012; Giovas, Kamenov, Fitzpatrick, & Krigbaum, 2016; Keller, Regan, Lundstrom, & Bower, 2016; Lamb, Evans, Buckley, & Appleby, 2014; Millard et al., 2014; Montgomery, Evans, Chenery, Pashley, & Killgrove, 2010; Price, Frei, Bäckström, Frei, & Ingvarsson‐Sundstrom, 2017; Sharpe et al., 2016; Shaw, Montgomery, Redfern, Gowland, & Evans, 2016; Smits, Millard, Nowell, & Graham Pearson, 2010; Turner, Kamenov, & Kingston, 2009; Valentine, Kamenov, & Krigbaum, 2008). Previous research has clearly demonstrated that among pre‐metallurgical populations, endogenous Pb in skeletal tissues predominantly reflects uptake of geological Pb through local environmental exposure, whereas populations exposed to anthropogenic Pb possess generally higher skeletal Pb concentrations and Pb isotope compositions that reflect the sources of ores used in metal production (Budd et al., 2004; Budd, Montgomery, Evans, & Barreiro, 2000; Evans et al., 2018; Kamenov & Gulson, 2014; Millard et al., 2014; Montgomery et al., 2005, 2010). Anthropogenic Pb exposure tends to overprint the relatively minor contribution of geogenic Pb to the overall pool of Pb in the body (Gulson, 2012; Kamenov & Gulson, 2014; Kamenov, Lofaro, Goad, & Krigbaum, 2018; Millard et al., 2014; Montgomery et al., 2010). Given the potential long‐distance transport of Pb‐bearing ores and the typically widespread circulation of metal objects, skeletal Pb isotope ratios in populations with high levels of anthropogenic Pb exposure are more informative of Pb ore sources than of individual geographic origins. As such it is necessary to consider the cultural chronology of metallurgical traditions (e.g., Pb ore mining) and potential exposure to various other sources of anthropogenic Pb in the study region to properly interpret skeletal Pb isotope results.

## 2 MATERIALS AND METHODS

### 2.1 Materials

**Figure 2:**
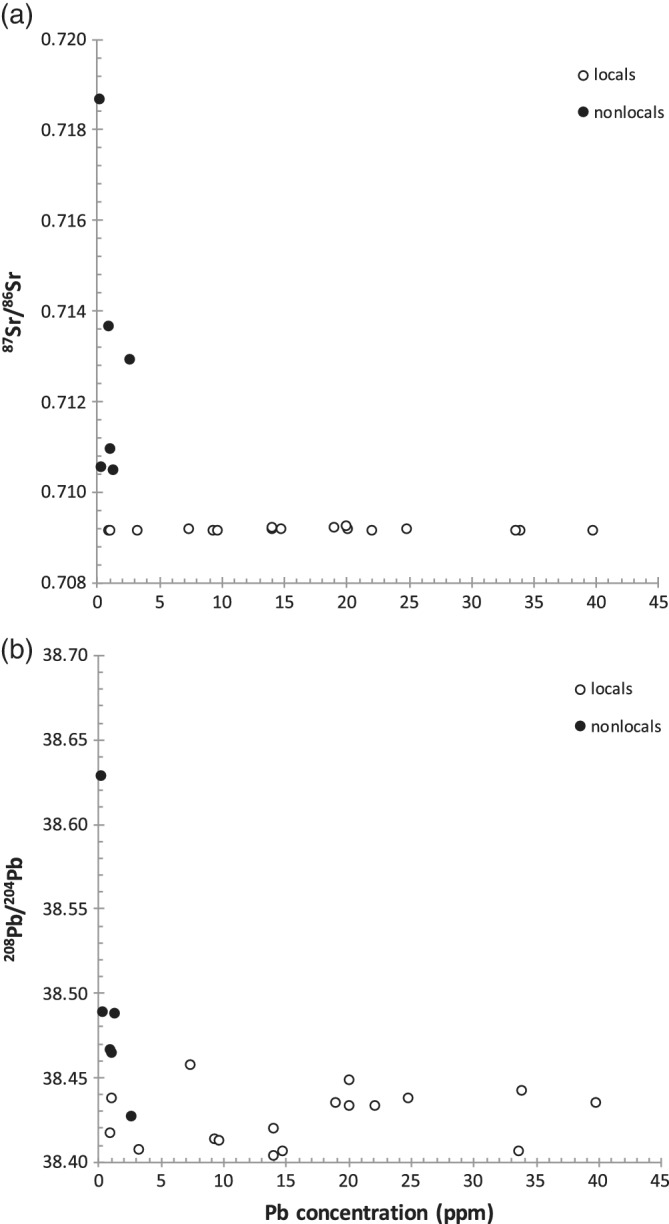
(a) Scatterplot of ^87^Sr/^86^Sr ratios versus enamel Pb concentrations; (b) ^208^Pb/^204^Pb ratios versus enamel Pb concentrations. ^87^Sr/^86^Sr data from Schroeder et al. (2009)

We analyzed dental enamel samples from 24 individuals from the Newton Plantation skeletal assemblage, one of the most well‐documented and well‐studied skeletal assemblages dating to the era of plantation slavery in the Caribbean (Handler & Corruccini, 1983; Handler & Lange, 1978). Dental samples selected for Pb isotope analyses (*n* = 24) mostly comprise first molars (M1) and were previously analyzed for strontium and oxygen isotope composition (Schroeder et al., 2009) and Pb concentrations (Schroeder et al., 2013). These previous results (Figure 2a) indicate the presence of at least seven first‐generation migrants from Africa with combined high (radiogenic) ^87^Sr/^86^Sr ratios (>0.710) and low Pb concentrations (<0.8 ppm). Several of these migrants also possessed intentionally modified anterior teeth. The present study presents new results of the analysis of Pb isotope compositions and Pb concentrations. The new Pb concentration data were obtained using a different method than the one used in Schroeder et al. in 2013 (Agilent 7500cx quadrupole ICP‐MS), but the results are comparable.

### 2.2 Analytical procedures

Each sample was rinsed several times with distilled water with 5 min of ultrasonication between each step. The samples were then dissolved in 2 M HCl, spiked with a ^205^Pb‐^235^U tracer solution of known concentration, dried down and redissolved in 1 M HBr. Lead from the samples was purified with a standard HBr‐HNO_3_ column chemistry using Eichrom anion resin, with each sample processed twice on the same column (Connelly & Bizzarro, 2009).

The purified Pb was loaded onto an outgassed zone‐refined Re ribbon with Si‐gel and H_3_PO_4_ and analyzed using a ThermoScientific™ Thermal Ionizing Mass Spectrometer equipped with nine Faraday detectors and one axial secondary electron multiplier‐ion counting (SEM‐IC) system. The natural isotopes of Pb were measured in static mode on Faraday detectors, whereas ^205^Pb was measured in the SEM‐IC. The Faraday‐SEM‐IC conversion factor was determined at the start of the run (after focusing the filament) by peak jumping ^204^Pb between the SEM‐IC and the Faraday cup H1. The ^206^Pb/^205^Pb ratio was used to determine the amount of Pb in each sample. The small contribution of natural Pb isotopes from the tracer to the sample is corrected according to the amount of ^205^Pb present in the analyses. Long‐term instrument performance is monitored by running the NIST981 Pb standard at the start and end of each measurement session. Blank contributions for this chemical procedure and loading the filaments are less than 0.5 pg, which is trivial given the amount of Pb from each sample.

## 3 RESULTS

**Figure 3:**
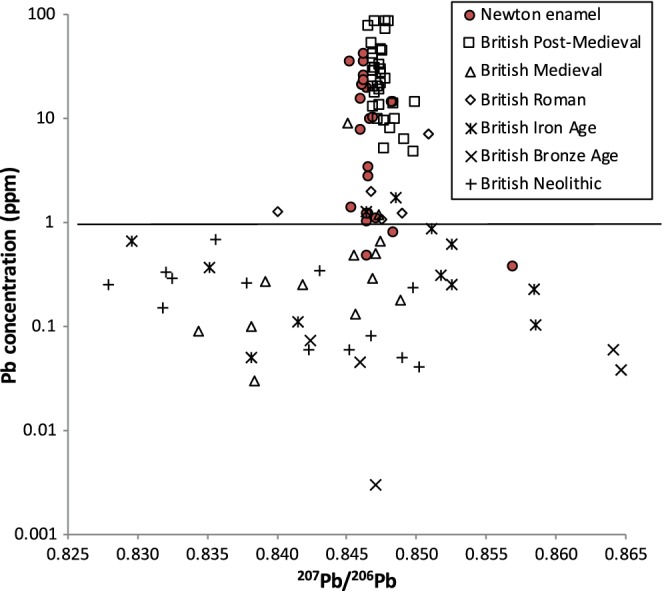
Enamel ^207^Pb/^206^Pb isotope ratios and concentrations from Newton Plantation, Barbados, compared to British enamel Pb data from different time periods (Millard et al., 2014; Montgomery et al., 2010). Note the logarithmic scale for concentration. The solid horizontal line is a lower limit of 0.87 ppm for “cultural” lead exposure determined by Millard et al. (2014)

**Figure 4:**
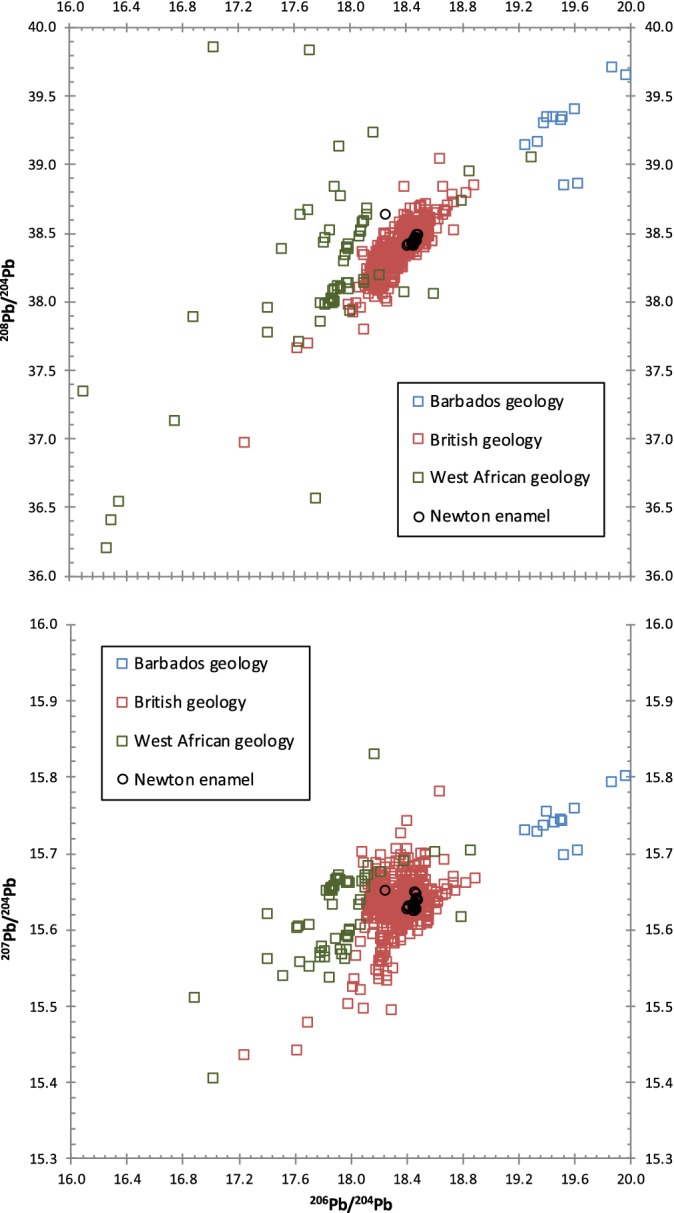
Comparison of Pb isotope data from locals and nonlocals at Newton Plantation, Barbados compared to geological Pb isotope data from Barbados, Britain, and West Africa. Data for Newton Plantation (this study); for Barbados basement rocks (Carpentier, Chauvel, & Mattielli, 2008); for British lead‐ore minerals (Fletcher, Swainbank, & Colman, 1993; Haggerty, Budd, Rohl, & Gale, 1996; Rohl, 1996; Scaife, Barreiro, McDonnell, & Pollard, 2001; Shepherd et al., 2009); for West African basement rocks and minerals (Dada, Adojoh, Rahaman, & Garba, 2015; Rademakers, Nikis, De Putter, & Degryse, 2018)

**Figure 5:**
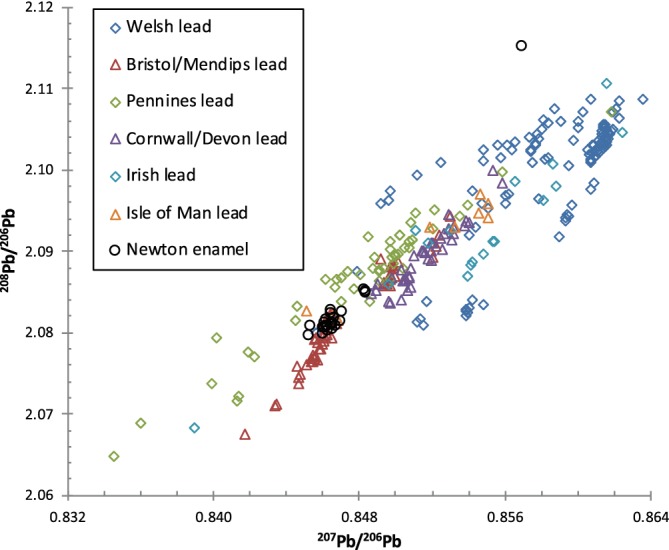
Enamel ^208^Pb/^206^Pb versus ^207^Pb/^206^Pb isotope ratios from Newton Plantation, Barbados, compared to geological Pb‐ore variation in Britain (Farmer, Eades, & Graham, 1999; Fletcher et al., 1993; Haggerty et al., 1996; Rohl, 1996; Scaife et al., 2001; Shepherd et al., 2009)

Relevant sampling information and Pb isotope results are listed in Table 1 and displayed in Figures 2, 3, 4, 5. Similar to previously reported results (Schroeder et al., 2013), the newly generated Pb concentrations are highly variable ranging from ~0.5 to 40 ppm (Table 2). Nonlocal individuals with elevated ^87^Sr/^86^Sr ratios (Figure 2a) have considerably lower (mean = 1.2 ppm) and less variable (range 0.4–2.7 ppm) Pb concentrations than locals (mean = 17.0 ppm, range 1–40 ppm). A Mann–Whitney test indicates that these differences are statistically significant (*U* = 6.5, *p* = .00086).

**Table 1:** Sampling information, Pb isotope results, and Pb concentrations from Newton Plantation, Barbados

Burial #	Age	Sex	^208^Pb/^206^Pb	^207^Pb/^206^Pb	^208^Pb/^204^Pb	^206^Pb/^204^Pb	^207^Pb/^204^Pb	Pb conc. (ppm)
N01	18–25	f	2.080	0.846	38.448	18.481	15.638	20.1
N02	>35	m	2.081	0.847	38.413	18.461	15.631	9.4
N07	4–8	?	2.082	0.846	38.438	18.466	15.627	24.9
N08	20–25	m	2.081	0.846	38.435	18.471	15.635	19.1
N10	15–20	f	2.080	0.845	38.442	18.485	15.626	34.0
N20	45–70	m	2.080	0.846	38.406	18.462	15.625	33.7
N22	18–23	m	2.081	0.846	38.433	18.468	15.627	20.1
N27	25–30	m	2.081	0.846	38.435	18.472	15.632	39.9
N33	>18	f	2.081	0.846	38.433	18.471	15.632	22.1
N34	20–25	m	2.080	0.846	38.406	18.467	15.625	14.9
N38	18–23	m	2.085	0.848	38.403	18.421	15.629	14.1
N42	9–11	?	2.081	0.847	38.413	18.456	15.632	9.8
N47	35–49	m	2.081	0.847	38.417	18.461	15.628	1.0
N48	20–35	m	2.082	0.847	38.437	18.465	15.634	1.2
N53	6–7	?	2.081	0.846	38.457	18.484	15.639	7.4
N54	25–35	f	2.080	0.847	38.407	18.462	15.630	3.3
N55	6–7	?	2.085	0.848	38.419	18.424	15.630	14.1
**N06**	21–40	?	2.085	0.848	38.400	18.418	15.625	0.8
**N19†**	>21	m	2.081	0.845	38.488	18.497	15.638	1.4
**N29**	>23	f	2.082	0.847	38.489	18.484	15.647	0.5
**N31**	>18	m	2.081	0.847	38.427	18.465	15.632	2.7
**N43**	16–23	m	2.083	0.847	38.464	18.468	15.634	1.2
**N52†**	30–35	f	2.115	0.857	38.628	18.264	15.651	0.4
**N56**	20–30	f	2.082	0.847	38.466	18.472	15.648	1.1

*Note*: Nonlocals are marked in bold; individuals with intentional dental modifications are noted with †.

**Table 2:** Summary statistics of Pb isotope results and Pb concentrations from Newton Plantation, Barbados

		^208^Pb/^206^Pb	^207^Pb/^206^Pb	^208^Pb/^204^Pb	^206^Pb/^204^Pb	^207^Pb/^204^Pb	Pb conc. (ppm)
All	Mean	2.083	0.847	38.442	18.456	15.633	12.4
	Min	2.080	0.845	38.400	18.264	15.625	0.4
	Max	2.115	0.857	38.628	18.497	15.651	39.9
	*SD*	0.007	0.002	0.047	0.045	0.007	12.1
	2*SD*	0.014	0.005	0.094	0.091	0.015	24.2
Locals	Mean	2.081	0.847	38.426	18.463	15.631	17.0
	Min	2.080	0.845	38.403	18.421	15.625	1.0
	Max	2.085	0.848	38.457	18.485	15.639	39.9
	*SD*	0.002	0.001	0.017	0.017	0.004	11.5
	2*SD*	0.003	0.002	0.033	0.035	0.009	23.0
Nonlocals	Mean	2.087	0.848	38.480	18.438	15.639	1.1
	Min	2.081	0.845	38.400	18.264	15.625	0.4
	Max	2.115	0.857	38.628	18.497	15.651	2.7
	*SD*	0.012	0.004	0.073	0.081	0.010	0.8
	2*SD*	0.025	0.008	0.146	0.162	0.019	1.6

A comparison of Pb concentrations with Pb isotope ratios (Figure 2b) indicates that individuals with low enamel Pb concentrations display a wider range of Pb isotope ratios than individuals with higher Pb concentrations. This pattern of inversely correlated Pb concentrations and Pb isotope ratios is consistent with the long‐term trend in human enamel Pb data in Europe that has been termed cultural focusing (Montgomery et al., 2005; Figure 3) and suggests that samples from Newton Plantation with higher Pb concentrations and similar Pb isotope ratios are the result of (childhood) exposure to anthropogenic Pb sources (Schroeder et al., 2013).

All 24 samples possess Pb isotope compositions that are distinct from those reported for Barbadian geological materials (Figure 4) and do not fall on the trendline for modern North American (anthropogenic) Pb sources (Kamenov & Gulson, 2014). Overall, the Pb isotope compositions of the entire data set are fairly homogeneous (Table 2) with the exception of one extreme outlier (N52). Individual N52 had previously been identified as a first‐generation African migrant on the basis of her elevated ^87^Sr/^86^Sr ratio (0.71866), low Pb concentration (0.4 ppm), and the presence of intentional dental modification. Interestingly, the other six nonlocal (African‐born) individuals possess Pb isotope compositions that are indistinguishable from the local population at Newton Plantation.

## 4 DISCUSSION

The fact that the Newton Plantation population possesses Pb isotope ratios that are distinct from the underlying geology of the island of Barbados suggests that Pb skeletal burdens may have resulted from exposure to imported (anthropogenic) Pb sources rather than reflecting exposure to natural (geogenic) Pb sources on Barbados (Figure 4). As a British colony, British Pb sources are a likely source of anthropogenic Pb for the residents of Newton Plantation in the form of imported food, drink, serving wares, and equipment for processing rum (Schroeder et al., 2013). In Figure 5, we plot the Newton Plantation human enamel Pb isotope data versus a compilation of reported British Pb ore‐source isotope data. The vast majority of the Newton individuals, with the exception of the one previously mentioned extreme outlier (N52), fall within the range of reported Pb isotope variation for Britain and are quite similar in particular to postmedieval human Pb isotope values from Britain (Figure 3). This indicates that the enamel Pb isotope signals of the Newton Plantation locals may reflect childhood exposure to anthropogenic (British) Pb that has overprinted the naturally low levels of Pb contributed by exposure to Barbadian geological Pb sources. It is worth noting that the outlier (N52) has Pb isotope ratios that fall completely out of the range of British Pb isotope compositions.

Turning to the nonlocal individuals, the seven individuals previously identified as African‐born (with high Sr isotope ratios and low Pb concentrations) are expected to possess Pb isotope signatures characteristic of the location(s) of their natal origins in Africa. However, six out of the seven nonlocals possess Pb isotope ratios that are indistinguishable from the local population, and by extension also fall within the range of British Pb sources. There are several, non‐mutually exclusive mechanisms that may account for these observed patterns in the data.

First, this could simply result from overlap in the ranges of Pb isotope variation (Figure 4) between Pb sources in Britain (reflected in the enamel Pb isotope ratios of the local population) and in West Africa (reflected in the enamel Pb isotope ratios of six of the nonlocals). Unfortunately, it is not possible to further constrain the potential origins of these individuals on the basis of the Pb isotope data alone, as the spatial variation of Pb isotopes in Africa is not well characterized. To our knowledge, little or no human Pb isotope data from Africa are available at this time for comparative analysis. However, given the documented geological complexity of West Africa, we would expect a high degree of spatial variation in both geological and bioavailable Pb isotopes in this region of the world. As such, it is not likely that the six nonlocal (African‐born) individuals would possess highly diverse geographic origins evidenced by their heterogeneous strontium isotope ratios and yet simultaneously also have nearly identical enamel Pb isotope ratios.

Second, it is possible given the long history of interaction and colonialism that West Africans (i.e., born and raised in Africa) in the 17th–18th century could have been exposed to technological (anthropogenic) lead imported or traded from a European source, although there is no direct evidence that this applies to the first‐generation Africans identified at Newton.

**Figure 6:**
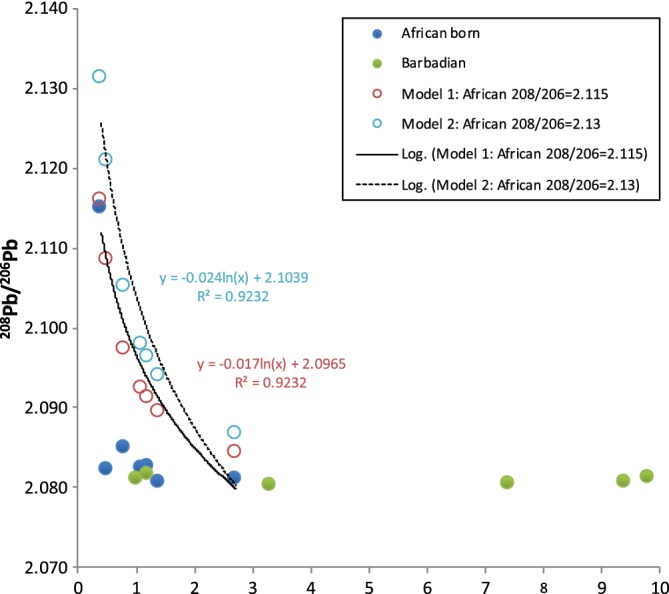
Mixing model illustrating the effects of increasing contributions of anthropogenic Pb on the Pb isotope ratios of nonlocal (African‐born) individuals from Newton Plantation, Barbados. Filled symbols are enamel ^208^Pb/^206^Pb isotope ratios plotted against Pb concentrations. Empty symbols are modeled values using the ^208^Pb/^206^Pb and Pb concentration of the highest outlier to represent a hypothetical African end member and the average ^208^Pb/^206^Pb of the local population to represent the other end member. Modeled Pb concentrations were estimated by subtracting the background African Pb of the highest outlier (0.41 ppm) from the measured Pb concentrations for each nonlocal. Modeled (local) anthropogenic Pb exposure ranges from <0.1 to 2.3 ppm

Third, the homogenous Pb isotope ratios in the Newton nonlocals reflect contamination from anthropogenic Pb sources on Barbados. As we have found no reported cases of in vivo Pb contamination of fully mature dental enamel in the biomedical literature, postmortem contamination (diagenesis) seems to be the most parsimonious explanation. In fact, previous research has identified postmortem alteration of Pb isotope ratios in archaeological skeletal remains in diverse geographical and cultural contexts (Chiaradia et al., 2003; Dudás et al., 2016) including in the Caribbean region (Giovas et al., 2016). Perhaps counter‐intuitively, individuals with the lowest Pb concentrations in their dental enamel (purportedly reflecting geogenic, as opposed to anthropogenic, Pb sources) are the most susceptible to alteration of their Pb isotope compositions (Kamenov et al., 2018). This process is illustrated with a simple mixing model (Figure 6) demonstrating that even minor contributions of non‐biogenic Pb can significantly alter the Pb isotope ratios measured in archeological dental enamel. This contrasts sharply with strontium with typical contributions in human skeletal remains (~200–500 ppm) that are several orders of magnitude greater than the enamel Pb concentrations observed in the Newton nonlocal data set (0.4–2.7 ppm). The one nonlocal individual with a distinct Pb isotope composition may have preserved an authentic biogenic (African) Pb signal in her dental enamel. The exact mechanisms accounting for the observed variation in Pb contamination are not known but previous research has implicated multiple variables such as burial depth, burial soil chemistry, and soil particle size (e.g., Chiaradia et al., 2003; Giovas et al., 2016).

Overall, we conclude that Pb isotope measurements are not very useful for human provenance studies for the Newton population in particular and that considerable caution is merited in the application of Pb isotope analysis in other early modern contexts given the potential susceptibility of dental enamel with low Pb concentrations to contamination with modern or past anthropogenic Pb. Nevertheless, the finding that the locally born fraction of the Newton population possesses both high Pb concentrations and enamel Pb isotope ratios that match the Pb isotope composition of British (Bristol/Mendips) Pb ore sources is still valid and most likely reflects exposure to high levels of anthropogenic Pb from early childhood at the Newton Plantation, with devastating health consequences for the population.

The health impacts of lead were common though poorly understood in British colonies in North America and the West Indies during the 17th and 18th centuries (Aufderheide et al., 1985; Corruccini et al., 1987; Handler et al., 1986; Schroeder et al., 2013). Physicians frequently described in the medical literature a mysterious illness that was variably referenced by such terms as “dry bellyache,” “dry gripes,” “West Indian dry gripes,” and “nervous colick,” among others (Handler et al., 1986, p. 408). Characterized by debilitating abdominal cramps, severe constipation without diarrhea, and weakness or paralysis of the limbs, this illness was widely known to be endemic among the white populations and descriptions are consistent with the clinical manifestations of lead poisoning (Handler et al., 1986).

Although the illness was very rarely mentioned for African populations, Handler and colleagues provide strong inferential evidence that the enslaved also suffered from the effects of lead that went unrecognized by early West Indian physicians (Corruccini et al., 1987; Handler et al., 1986). Ingestion of food tainted by imported lead is widely implicated in cases of lead poisoning on plantations throughout the British colonies (Aufderheide et al., 1985; Aufderheide, Neiman, Wittmers, & Rapp, 1981; Wittmers, Aufderheide, Rapp, & Alich, 2002), though Handler and colleagues have argued that the enslaved would have low access to many household items such as pewter, powders, flours and other sources that commonly sickened white households and domestic servants. Rather, they implicated rum distillation and consumption as the most likely sources of lead on Caribbean plantations, notably on Barbados, where skeletal and dental evidence has been used to attest to high lead levels in enslaved rum producers (Corruccini et al., 1987; Handler et al., 1986; Schroeder et al., 2013). The high to extreme lead values observed at Newton during infancy to early childhood, along with near absence of developmental features that were predicted with rum consumption, seem to suggest greater complexity in routes of exposure (Shuler & Schroeder, 2013). Epidemiological studies of contemporary potters and their families on Barbados, for example, have shown that elevated blood values and health impacts (e.g., tremors) can result from lead dust transported from work areas to households, consequently affecting children (Koplan, Wells, Diggory, Baker, & Liddle, 1977).

## 5 CONCLUDING REMARKS

This study presents the results of Pb isotope analyses of human dental enamel from the 17th/18th century enslaved African population of Newton Plantation, Barbados. The Pb isotope data are interpreted in the context of archaeological, bioarchaeological, historical, and previously generated strontium isotopic data (Schroeder et al., 2013). In contrast to expectations, the Pb isotope data neither mirrored nor complemented the Sr isotope data and only revealed the presence of one statistical outlier (whereas the Sr isotope data had indicated the presence of seven nonlocal individuals). Furthermore, none of the measured individuals (including the local population) possessed Pb isotope compositions that were comparable to Barbadian geological sources, and six out of seven African‐born individuals have Pb isotope signatures that are broadly similar to the locals at Newton Plantation.

These combined results indicate that (a) the local population at Newton Plantation possesses Pb isotope ratios indicative of exposure to high levels of anthropogenic Pb likely derived from British Pb sources, and (b) owing to their low Pb concentrations, the majority of the nonlocal population (six out of seven) possesses Pb isotope ratios that may have been significantly altered by diagenetic contamination. In light of this, future research is needed to (a) develop effective methods for identifying diagenetic alteration of archaeological enamel samples via, for example, Fourier‐transform infrared spectroscopy to check for changes in bioapatite crystallinity or trace element analysis to detect the uptake of other diagenetic contaminants; and (b) remove, or mediate the effects of, exogenous (non‐biogenic) Pb via more aggressive chemical pretreatment and leaching of enamel samples.

In summary, the Pb isotope analyses provided limited new information concerning the provenance of the nonlocal individuals at Newton Plantation but provided important new insights into the sources and extent of anthropogenic Pb exposure of the local Barbadian population. These new insights highlight the previously noted serious health effects of Pb poisoning in colonial contexts and indicate that continued studies of Pb isotope compositions and Pb concentrations of human skeletal remains may further contribute to disentangling the complex and dynamic processes of colonialism, enslavement, culture contact, occupation, and health in colonial contexts.

## Data Availability

The authors confirm that the data supporting the findings of this study are available within the article [and/or] its supplementary materials.
